# Crosstalk Between the NLRP3 Inflammasome/ASC Speck and Amyloid Protein Aggregates Drives Disease Progression in Alzheimer’s and Parkinson’s Disease

**DOI:** 10.3389/fnmol.2022.805169

**Published:** 2022-02-03

**Authors:** Jonathan Hulse, Kiran Bhaskar

**Affiliations:** ^1^Department of Molecular Genetics and Microbiology, University of New Mexico, Albuquerque, NM, United States; ^2^Department of Neurology, University of New Mexico, Albuquerque, NM, United States

**Keywords:** inflammasomes, NLRP3, ASC, Alzheimer’s disease, Parkinson’s disease, protein aggregation, neuroinflammation, autophagy-lysosomal degradation

## Abstract

Two key pathological hallmarks of neurodegenerative diseases, including Alzheimer’s disease (AD) and Parkinson’s disease (PD), are the accumulation of misfolded protein aggregates and the chronic progressive neuroinflammation that they trigger. Numerous original research and reviews have provided a comprehensive understanding of how aggregated proteins (amyloid β, pathological tau, and α-synuclein) contribute to the disease, including driving sterile inflammation, in part, through the aggregation of multi-protein inflammasome complexes and the ASC speck [composed of NOD-, LRR-, and pyrin domain-containing protein 3 (NLRP3), Apoptosis-associated speck-like protein containing a C-terminal caspase activation and recruitment domain (ASC), and inflammatory caspase-1] involved in innate immunity. Here, we provide a unique perspective on the crosstalk between the aggregation-prone proteins involved in AD/PD and the multi-protein inflammasome complex/ASC speck that fuels feed-forward exacerbation of each other, driving neurodegeneration. Failed turnover of protein aggregates (both AD/PD related aggregates and the ASC speck) by protein degradation pathways, prionoid propagation of inflammation by the ASC speck, cross-seeding of protein aggregation by the ASC speck, and pro-aggregatory cleavage of proteins by caspase-1 are some of the mechanisms that exacerbate disease progression. We also review studies that provide this causal framework and highlight how the ASC speck serves as a platform for the propagation and spreading of inflammation and protein aggregation that drives AD and PD.

## Introduction

Alzheimer’s disease (AD) is the leading cause of dementia and is in the top 10 causes of death worldwide (Lane et al., 2018; Breijyeh and Karaman, 2020). It is characterized clinically by progressive memory loss, functional, and cognitive impairment (Lane et al., 2018; Breijyeh and Karaman, 2020). Three key components of the pathophysiology of AD include the accumulation of extracellular Amyloid-β (Aβ) plaques, intracellular hyperphosphorylated tau neurofibrillary tangles, and chronic neuroinflammation (Lane et al., 2018; Breijyeh and Karaman, 2020). Early stages of the disease classically affect the hippocampus and then progress to diffuse cerebral cortex involvement (Lane et al., 2018; Breijyeh and Karaman, 2020).

Parkinson’s disease (PD) is the second leading cause of neurodegeneration. PD is primarily a movement disorder that classically presents with bradykinesia, cogwheel rigidity, resting tremor, and postural imbalance (Cacabelos, 2017). The pathophysiology of PD is characterized by the accumulation of intracellular α-synuclein (α-syn) aggregates known as Lewy bodies and chronic inflammation with degeneration of dopaminergic neurons in the basal ganglia (Cacabelos, 2017).

There are numerous other neurodegenerative diseases that share commonalties in their pathogenesis including amyotrophic lateral sclerosis (ALS) (Liu and Wang, 2017), Huntingtin’s disease (Lois et al., 2018), and the range of tauopathies including supranuclear palsy, frontotemporal dementia (FTD), corticobasal degeneration, FTD and parkinsonism linked to chromosome 17, and others (Arendt et al., 2016). All of these are characterized by misfolded protein aggregation and chronic neuroinflammation. Though each condition is characterized by unique disease-relevant proteins that affect specific brain regions, each condition shares several commonalities including: protein misfolding and aggregation into β-sheet rich fibrillar aggregates, maladaptive innate immune responses with chronic progressive neuroinflammation, defective protein-quality control and degradation, substantial neuronal cell loss, and some form of cognitive or functional impairment (Gan et al., 2018; Ciccocioppo et al., 2020). This review will primarily focus on literature relevant to AD and PD though it is likely that much of the discussion can be extended to these other neurodegenerative proteinopathies.

Having shown by many studies to be primary drivers of the disease, pathologic protein aggregates have been targeted as potential therapy for AD/PD. For example, the AN1792 vaccine, and the monoclonal antibodies Bapineuzumab, Solanezumab, Gantenerumab, Crenezumab, and BAN2401 are clinical candidates for AD (Schneider et al., 2014). Recently, the FDA has conditionally approved Aduhelm^®^, an immunotherapy against amyloid pathology, requiring post-approval monitoring due to controversy among the scientific community about its effectiveness and potential side effects, especially amyloid-related imaging abnormalities (ARIA) linked to cerebral edema and intracerebral hemorrhage (Tampi et al., 2021). In the past two decades, many therapeutics have only targeted protein build-up in AD and PD, failing to consider other co-etiologies. There is consensus that this might have contributed to significant failure rate (>99%) in translating pre-clinical studies to clinical utility. Among many co-occurring pathological signatures, neuroinflammation may play a more critical role in disease progression than was previously emphasized by the amyloid cascade hypothesis, which viewed neuroinflammation as a byproduct of amyloid toxicity. In the last decade, an inflammation-based hypothesis has gained favor to explain the mechanism of neurodegenerative disease progression whereby inflammation may serve as an inciting event early in the disease that contributes to protein misfolding and aggregation which reinforces neuroinflammation in a positive-feedback manner, ultimately leading to neurodegeneration (McGeer and McGeer, 2013). Finally, recent genome-wide association studies (GWAS) have identified several genes in the immune pathways showing strong association to sporadic AD (e.g., *CD33, TREM2* etc.) and PD (e.g., *BST1, HLA*) (Billingsley et al., 2018; Neuner et al., 2020).

Many studies have shown a link between these misfolded protein aggregates and inflammation. The nod-like receptor family pyrin domain containing 3 (NLRP3) inflammasome is one of the most well characterized sensors of danger signals in cells, most associated with neurodegenerative diseases, and serves as a connection point between neuronally-derived misfolded protein aggregates and the damaging neuroinflammation associated with neurodegeneration (de Alba, 2019; Swanson et al., 2019). The NLRP3 inflammasome is a component of the innate immune system that responds to pathogen-associated molecular patterns (PAMPs) and damage-associated molecular patterns (DAMPs) in a pattern recognition receptor (PRR)-dependent manner (de Alba, 2019; Kelley et al., 2019; Swanson et al., 2019). It is a multi-protein complex composed of NLRP3, which serves as an intracellular sensor for various danger signals, the apoptosis-associated speck-like protein containing a caspase activation and recruitment domain or CARD domain (ASC) which serves as an adaptor protein, and caspase-1 which carries out the enzymatic cleavage of interleukin (IL)-1β, IL-18, and gasdermin-D among others (de Alba, 2019; Swanson et al., 2019). In settings of acute inflammation, the NLRP3 inflammasome components and its byproducts undergo clearance to avoid excessive or sustained activation of inflammatory signaling upon resolution of the inflammatory stimuli.

**FIGURE 1:**
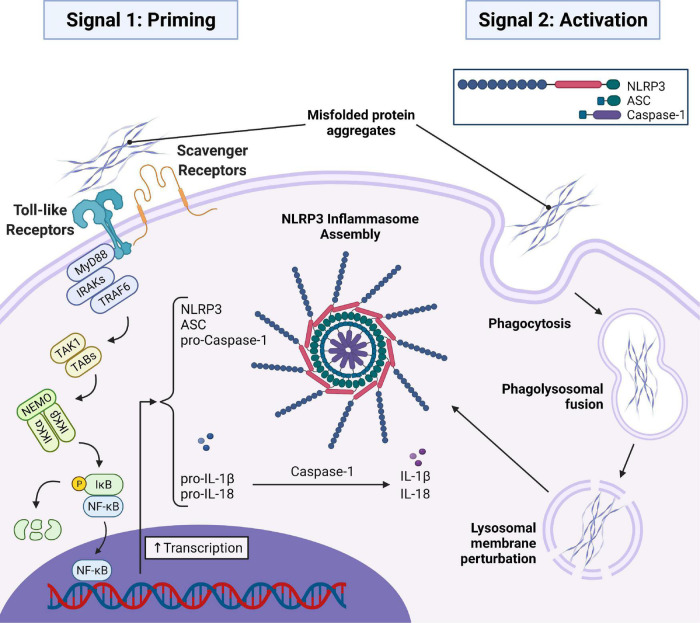
AD and PD related misfolded protein aggregates (Aβ, tau, and α-syn) initiate signal 1 and signal 2 required for assembly of the NLRP3 inflammasome and subsequent ASC speck. In signal 1, misfolded protein aggregates bind to toll-like and scavenger receptors on the microglial cell surface initiating a MyD88-dependent signaling transduction event resulting in the translocation of NF-κB into the nucleus to initiate the transcription of important inflammasome components and the immature forms of the inflammatory cytokines pro-IL-1β and pro-IL-18. In signal 2, misfolded protein aggregates are taken into the cell via phagocytosis and trafficked to the lysosome where they disrupt the lysosomal membrane and trigger assembly of the inflammasome. Oligomerization of NLRP3 then recruits ASC which oligomerizes with NLRP3 via homotypic interactions between PYD regions of both proteins. Pro-caspase-1 is then recruited to the oligomerized ASC via homotypic interactions between CARD regions. Dimerization of pro-caspase-1 triggers an autolytic cleavage event producing two active caspase-1 enzymes that can mature pro-IL-1β and pro-IL-18 into the mature IL-1β and IL-18. MyD88, myeloid differentiation primary response gene 88; IRAK, IL-1 receptor-associated kinase; TRAF6, TNF receptor associated factor 6; TAK1, transforming growth factor-β-activated kinase-1; TAB, TAK1 binding proteins; NEMO, NF-κB essential modulator; IKKα/β, Inhibitor of NF-κB Kinase; IκB, Inhibitor of NF-κB; NF-κB, nuclear factor-kappaB; IL-1β/18, interleukin-1β/18.

Aβ aggregates have been shown to activate the transcription of inflammatory cytokines and proteins required for the assembly of the NLRP3 inflammasome through toll-like and scavenger receptor signaling pathways (Udan et al., 2008; Liu et al., 2020). Activation of the receptor recruits myeloid-differentiation primary response gene 88 (MyD88) to the cytoplasmic domain of the receptor. MyD88 then recruits IL-1 receptor-associated kinase (IRAK) proteins, namely IRAK4 and IRAK1, which are sequentially phosphorylated and can activate TNF receptor-associated factor 6 (TRAF6). TRAF6 is an E3-ligase that K63-pulyubiquitinates itself and NF-κB essential modulator (NEMO), recruiting transforming growth factor-β-activated kinase 1 (TAK1) and TAK1 binding proteins (TABs) to phosphorylate the inhibitor of NF-κB kinase (IKK) complex, which then phosphorylates the inhibitor of NF-κB (IκB) protein. This allows for the release of NF-κB from its inhibitory IκB complex, which is subsequently degraded, so that NF-κB can translocate to the nucleus and initiate the transcription of NLRP3, ASC, pro-caspase-1, pro-IL-1β, and pro-IL-18 (Kawai and Akira, 2007). This is referred to as the priming step, or signal 1, for inflammasome activation (Figure 1). Aβ aggregates have also been shown to directly trigger the activation step, or signal 2, for NLRP3 inflammasome assembly (Figure 1) in microglia (Halle et al., 2008). The mechanism by which this occurs is not entirely clear but has been demonstrated to involve the phagocytosis of the Aβ aggregates by microglia which then escape lysosomal degradation through damage and destabilization of the lysosome (Halle et al., 2008), upon which they can trigger NLRP3 inflammasome and ASC speck assembly in a manner similar to large crystals (Martinon et al., 2006). Whether or not NLRP3 inflammasome activation by protein aggregates can occur independent of lysosomal damage is unclear, though dysregulation of mitochondrial homeostasis by α-syn has been shown to be another contributor to inflammasome activation (Xie and Chung, 2012; Zhong et al., 2018). Similar findings have demonstrated identical triggering of both the priming (signal 1) and activation (signal 2) steps of the NLRP3 inflammasome by pathological phosphorylated tau aggregates by us (Jiang et al., 2021) and others (Stancu et al., 2019). Additionally, α-syn aggregates have also been shown to trigger both signals (Codolo et al., 2013; Gordon et al., 2018).

NLRP3 oligomerizes upon activation by intracellular protein aggregates. This oligomerization then recruits monomeric ASC which oligomerizes with NLRP3 via homotypic interactions between the pyrin domain (PYD) of ASC and the PYD of NLRP3 (de Alba, 2019; Swanson et al., 2019). Upon aggregation of ASC with NLRP3, pro-caspase-1 is recruited and binds to ASC via homotypic CARD-CARD interactions between the two proteins. The assembly of these three proteins forms the basic functional unit of the inflammasome complex. When two monomers of pro-caspase-1 dimerize on the ASC scaffolding, they are activated through their intrinsic auto-cleavage capacity yielding two functional caspase-1 enzymes that can then activate numerous other proteins involved in the inflammatory cascade (de Alba, 2019; Swanson et al., 2019). In addition to forming the basic inflammasome complex, NLRP3 inflammasome activation can also result in the formation of a dense structure ∼1 μM in diameter referred to as the ASC speck (de Alba, 2019; Swanson et al., 2019).

The ASC speck is a supramolecular aggregate of the inflammasome complex composed primarily of ASC that serves as a signal amplification platform for enhanced cytokine maturation by caspase-1 (Dick et al., 2016). Upon inflammasome activation and recruitment of ASC to oligomerized NLRP3, ASC continues to aggregate via homotypic interactions between its PYD region into large helical fibrils. Numerous ASC fibrils are then cross-linked via homotypic interactions on the ASC-CARD region into a dense ASC speck. The ASC speck continues to recruit pro-caspase-1 to its surface and functions via the same mechanism as the basic inflammasome complex at a larger scale (de Alba, 2019).

Recent studies have implicated an important role for the NLRP3 inflammasome and the ASC speck in the propagation and spreading of neuroinflammation and misfolded protein aggregation in neurodegenerative diseases and are reviewed here. The goal of this review is not to expand on the inflammasome in neurodegenerative disease, but to highlight our current understanding of this emerging paradigm of the inflammasome/ASC speck as an active contributor to AD/PD pathology through three key topics: (1) How failed protein aggregate clearance and regulation mechanisms contribute to the accumulation of microglial inflammasome protein aggregates and neuronally derived (AD/PD related) misfolded protein aggregates in parallel; (2) How prionoid activity, cross-talk, and cross-seeding of these aggregates contributes to a viscous feed-forward cycle resulting in AD/PD disease progression; and (3) How targeting the microglial inflammasome protein aggregates for therapeutics shows potential in breaking this cycle for the treatment of AD and PD. We encourage readers to refer to other reviews (Pereira et al., 2019; Venegas and Heneka, 2019; Hanslik and Ulland, 2020; Tan et al., 2020) which describe inflammasomes and the possible role that they play in the development and progression of neurodegenerative diseases such as AD and PD. Because microglia are the primary immune cells of the brain, this review will focus on the role of microglia in neuroinflammatory processes relevant to AD and PD.

## Regulation of Inflammasome and ASC Speck Assembly by Post-translational Modifications

### Phosphorylation and Dephosphorylation

Phosphorylation and dephosphorylation are the most common post-translational modifications (PTM) and are regulated by the interplay of hundreds of kinases and phosphatases. The regulation of NLRP3 inflammasomes have been extensively reviewed in Gong et al. (2018). Phosphorylation or dephosphorylation of various amino acids on NLRP3 are important for directly regulating the oligomerization of NLRP3 as well as the NLRP3-ASC interaction. One study showed that phosphorylation of NLRP3 at serine (S) 5 prevents the accidental oligomerization of NLRP3 and that phosphatase (PP)2A activity is important for NLRP3 activation (Stutz et al., 2017). Another study showed that dephosphorylation at tyrosine (Y) 861 by Protein Tyrosine Phosphatase Non-receptor Type 22 (PTPN22) allows for enhanced NLRP3 oligomerization indicating that tyrosine phosphorylation is an important regulator of aberrant inflammasome activation (Spalinger et al., 2016).

Additionally, phosphorylation can indirectly regulate NLRP3 inflammasome priming, inhibition, and degradation through the delicate balance between ubiquitination and deubiquitination of NLRP3. C-Jun N-terminal kinases (JNK)-mediated phosphorylation of NLRP3 at S194 is a critical priming step for inflammasome activation by promoting NLRP3 deubiquitination and oligomerization (Song et al., 2017). Conversely, Protein Kinase A (PKA)-mediated phosphorylation of NLRP3 at S291 (Guo et al., 2016) and S295 (Mortimer et al., 2016) can act as a critical brake to stop NLRP3 activation and promotes its ubiquitin-mediated degradation. Similarly, the Src family kinase LYN-mediated phosphorylation of NLRP3 at Y918 also promotes its ubiquitin-mediated degradation by the proteasome to negatively regulate inflammasome activatability (Tang et al., 2021).

Phosphorylation and constitutive interaction of ASC with IKKα prior to inflammasome activation is important for its negative regulation and sequestration in the nuclear compartment (Martin et al., 2014). Phosphorylation by IKKi is important for its translocation to the cytosol upon inflammasome priming, which then recruits the phosphatase PP2A to inhibit the negative regulatory kinase activity of IKKα (Martin et al., 2014). ASC phosphorylation by the TAK1-JNK pathway and Spleen Associated Tyrosine Kinase (SYK)-Protein-Tyrosine Kinase 2-Beta (PYK2) pathway in numerous models have been identified as contributing to ASC oligomerization during inflammasome activation and phosphorylation at Y144 in mouse ASC (human Y146) is critical for ASC Speck formation (Hara et al., 2013; Okada et al., 2014; Chung et al., 2016). A recent study has also demonstrated that Y60 and Y137 phosphorylation is also critical for ASC oligomerization and ASC speck formation and that ASC dephosphorylation may also play a critical role in promoting ASC speck assembly (Mambwe et al., 2019).

### Ubiquitination and Deubiquitination

Protein ubiquitination is a complex PTM mediated by a large variety of enzymes. In general, the addition of ubiquitin to a protein is regulated by an E3 ubiquitin ligase and its removal is regulated by deubiquitinases (Komander and Rape, 2012; Zheng and Shabek, 2017; Grumati and Dikic, 2018). The modification of proteins by the ubiquitin system is a tightly regulated balance between these classes of enzymes (Clague et al., 2019). Regulation of the NLRP3 inflammasome by the ubiquitin system occurs primarily through the function of numerous E3 ubiquitin ligases on each sub-component of the inflammasome and are reviewed extensively in Lopez-Castejon (2020).

Multiple E3 negative regulators that affect NLRP3 levels or activation have been identified including *FBXL2* (Han et al., 2015), *PARKIN* (Kang et al., 2016), and *TRIM31* (Song et al., 2016), which provide a signal for proteasomal degradation, *MARCH7* (Yan et al., 2015), which provides a signal for degradation by autophagy, and *ARIH2* (Kawashima et al., 2017) and *CUL1* (Wan et al., 2019), which bind and ubiquitinate NLRP3 to maintain its inactive state. Positive regulation of NLRP3 by the ubiquitin system has been observed with the interplay of numerous E3 enzymes including *PELI2* (Humphries et al., 2018), *TRAF6* (Xing et al., 2017), and *TRIM33* (Weng et al., 2014), the E2 enzyme *UBC13* (Ni et al., 2021), as well as the deubiquitinating enzymes *BRCC3/ABRO1* (Py et al., 2013; Ren et al., 2019), *USP7* and *USP47* (Lopez-Castejon et al., 2013), and *UCHL5* (Kummari et al., 2015). Negative regulation of the NLRP3 and AIM2 inflammasomes by ubiquitination of ASC promoting proteasomal degradation has also been shown by the *TRAF6* E3 enzyme (Chiu et al., 2016) which, interestingly, has been shown to be a positive regulator of NLRP3. Taken together, these studies indicate competing roles of the enzyme depending on the activating stimuli and cellular environment. Positive regulators that are required for the activation of ASC include the *TRAF3* (Siu et al., 2019) and *LUBAC* E3 enzymes (Elliott et al., 2014; Rodgers et al., 2014; Gurung et al., 2015) as well as the *USP50* deubiquitinating enzyme (Lee et al., 2017). Regulation of caspase-1 by the ubiquitin system has been implicated in numerous E3 IAPs (inhibitor of apoptosis proteins) as well as the *LUBAC* member *SHARPIN*, however, these mechanisms appear to be complex and are not yet well understood (Lopez-Castejon, 2020). While mutations in PARKIN have been directly implicated in the pathogenesis of familial PD (Dawson and Dawson, 2010), it is not well understood how integral each of these enzymes are in orchestrating the inflammasome response that contributes to AD/PD.

Much of our understanding of the role of the ubiquitin system on regulating the inflammasome focuses on regulating the activation and formation of the inflammasome complex through the regulation of its subcomponents. Some research has indicated crosstalk between the inflammasome and ubiquitin system. For instance, Eldritch et al. showed that caspase-1 can inactivate the E2 enzyme *UBE2L3* which decreases the proteasomal-tagging ubiquitination of pro-IL-1β, thus allowing for an enhanced IL-1β inflammatory response (Eldridge et al., 2017).

### Other Post-translational Modifications

Other PTMs that have been shown to regulate the inflammasome but are less commonly described include *S*-nitrosylation, SUMOylation, ADP-ribosylation, and proteolytic processing of the inflammasome components, many of which are reviewed in Yang et al. (2017). *S*-nitrosylation by nitric oxide (NO) of NLRP3 can inhibit ASC oligomerization and inflammasome assembly and *S*-nitrosylation of caspases, including caspase-1, can inhibit its catalytic function to suppress IL-1β and IL-18 maturation and release (Kim et al., 1998; Hernandez-Cuellar et al., 2012; Mao et al., 2013). Interestingly, inflammasome activation tends to upregulate NO generation suggesting an autoregulatory mechanism (Yang et al., 2017). Recent studies have demonstrated that SUMOylation of NLRP3 by *UBC9*-directed *SUMO1* and *TRIM28*-directed *SUMO1–3* are important for inflammasome activation and that de-SUMOylation by the SUMO-specific protease *SENP3* can reduce inflammasome activation (Shao et al., 2020; Qin et al., 2021). ADP-ribosylation of NLRP3 by the *Mycoplasma pneumoniae* CARDS-toxin can trigger inflammasome activation for a robust inflammatory response (Bose et al., 2014). Enterovirus 71 protease 2A and 3C (Wang et al., 2015) as well as *Mycobacterium tuberculosis* ZMP1 protease (Master et al., 2008) inhibit NLRP3 inflammasome activity through proteolytic cleavage of NLRP3.

### There Are Numerous Strategies to Regulate ASC Speck Assembly

PTM of the NLRP3 inflammasome components is highly important for regulating the activation of the inflammasome. In fact, many PTMs are required for inflammasome assembly to occur (Yang et al., 2017). This highly complex and versatile process contributes to the distinct inflammatory states in response to a variety of damaging stimuli, as well as the cellular decision to either stop the inflammatory cascade or to progress to pyroptosis (Yang et al., 2017; Gong et al., 2018; Lopez-Castejon, 2020; Guo et al., 2021). The various PTM systems may thus be an attractive target for controlling the activation of the inflammasome for the management of many inflammatory conditions, including AD and PD. Regulation of inflammasome assembly can also occur more upstream of inflammasome proteins, such as through regulation of the NF-κB pathway (Afonina et al., 2017) or transcriptional repression by methylation (Yi, 2021). There are clearly numerous cellular strategies to regulate the formation of the NLRP3 inflammasome and ASC speck. Later, we will discuss how failure to regulate and degrade the NLRP3 inflammasome/ASC speck, once it has been assembled, may contribute to the progression of AD/PD.

## The Role of the ASC Speck in the Chronic Progressive Neuroinflammatory State in Alzheimer’s Disease and Parkinson’s Disease

### Propagation of Inflammation via the ASC Speck

**FIGURE 2:**
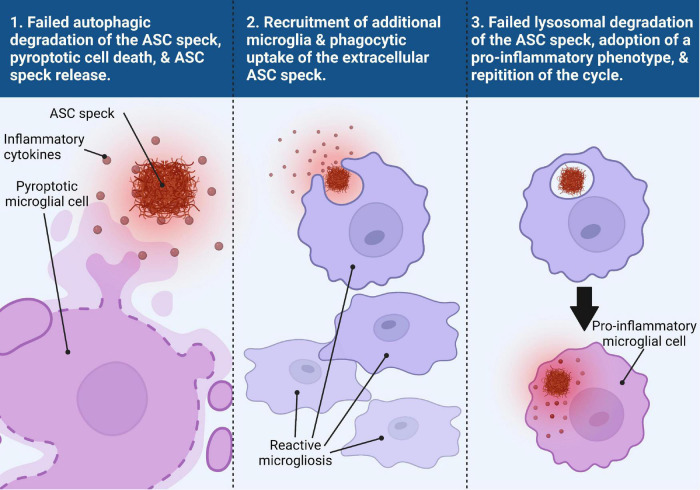
Trans-microglial propagation of inflammation via ASC speck. Upon activation and assembly of the ASC speck from the NLRP3 inflammasome in response to misfolded protein aggregates, the microglial cell fails to degrade the ASC speck via autophagic clearance mechanisms (panel 1). Persistence of the ASC speck can result in either a non-pyroptotic exocytosis of the ASC speck or pyroptotic cell death, resulting in the release of inflammatory cytokines and the ASC speck into the extracellular space (panel 1). The extracellular ASC speck remains functionally active and the presence of inflammatory cytokines helps to recruit additional reactive microglia to the site of inflammation (panel 2). The ASC speck can then be taken up by another microglial cell where it can be degraded by the lysosomes (panel 3). Failed degradation of the ASC speck and possible lysosomal damage induces the microglial cell to adopt a pro-inflammatory phenotype (panel 3). This microglial cell can then undergo the same process as the previous microglial cell, perpetuating a viscous inflammatory cycle.

Franklin et al. (2014) published a groundbreaking study on the extracellular activities of the ASC speck, identifying a mechanism for a prion-like propagation of inflammation from macrophage to macrophage in a paracrine fashion, that opened the door for a new paradigm in inflammasome-related pathology. They found that ASC speck formation upon activation of the NLRP3, NLRP1, NLRC4, and AIM2 inflammasomes preceded cell death via pyroptosis, upon which the speck is released into the extracellular space where it is still functionally active for the maturation of caspase-1 and IL-1β (Franklin et al., 2014). The extracellular ASC speck is also taken up by other phagocytic cells where it can either be degraded by the phagolysosomal pathway or behave like an exogenous danger signal by inducing lysosomal damage and further promote inflammasome activation in a similar manner to other fibrillar protein aggregates like Aβ and tau (Franklin et al., 2014). This work confirmed and expanded upon similar work that showed that NLRP3 inflammasome particles are secreted extracellularly where they can continue to mature caspase-1 in the extracellular space as well as within other macrophage cells upon phagocytosis of the particle (Baroja-Mazo et al., 2014). While extracellular ASC specks have been commonly seen in models of neurodegenerative disorders, not all microglia that form ASC specks undergo pyroptosis. It is possible that ASC specks may be “actively” released into the extracellular space in a manner that does not involve cell death though this has not been shown. Our recent studies have even suggested the presence of ASC specks in the cerebrospinal fluid of patients with AD and related tauopathies (Jiang et al., 2021). A model summarizing this process is shown in Figure 2.

In the context of neurodegenerative disease, this prion-like propagation of inflammation by the ASC speck is problematic because it contributes to the toxic, chronic progressive inflammatory state that plays a central role in neurodegeneration (Lee et al., 2019). Thus, prion-like pro-inflammatory activity of the ASC speck appears to be a maladaptive innate immune response. However, prion-like polymerization has been shown to be an evolutionarily conserved mechanism of signal transduction in innate immune defense (Cai et al., 2014) indicating that it must confer evolutionary benefits. Understanding how to better regulate this activity may be essential for development of therapeutics that limit the damaging effects of dysregulated inflammation in various diseases.

### Failed Degradation of the Inflammasome and ASC Speck

PTMs of the assembled inflammasome have been shown to be necessary for degradation of the inflammasome, including the ASC speck, via the autophagosome. Autophagy can negatively modulate inflammasome activity and pyroptosis through the degradation of PAMPs/DAMPS, degradation of the active inflammasome or ASC speck after PTM via the ubiquitin system, or degradation of the downstream products of inflammasome activity such as IL-1β and IL-18 (Guo et al., 2021).

It has been known for some time now that inflammasome activation triggers autophagy pathways in an inflammasome sensor-dependent (NLRP3-dependent) manner indicating an autoregulatory mechanism for resolution of inflammatory signaling. Inflammasomes co-localize with autophagosomes and lysosomes that contain inflammasome components (Shi et al., 2012). Inflammasomes are K63 polyubiquitinated and recruit the autophagic adaptor protein, p62, for delivery of the inflammasome to the autophagosome (Shi et al., 2012). It is currently not completely understood why these regulatory mechanisms fail in the setting of pyroptosis and chronic inflammatory states (Guo et al., 2021) where the ASC-speck can escape the clearance mechanism and become prionoid. The ASC speck’s prion-like structure is resistant to proteolytic degradation much like the misfolded protein aggregates in AD and PD (Franklin et al., 2014). Thus, failure of autophagic clearance mechanisms of the ASC speck results in a substantial lost opportunity for degradation that allows it to persist to the point that the inflammatory response becomes toxic.

Neurodegenerative diseases such as AD and PD are characterized by failure of neuronal protein degradation systems leading to the accumulation of misfolded protein aggregates (Ciechanover and Kwon, 2015; Jiang and Bhaskar, 2020). It is unclear if this is primarily due to down-regulation of these pathways due to environmental stressors, decreased activity due to cellular aging, or some other mechanism. Heneka et al. (2018) proposed that old age contributes to immuno-senescence of microglia resulting in an altered hyper-responsive inflammatory response that may predispose the brain to chronic inflammatory states. This alone may possibly contribute to the failure of these clearance mechanisms. Whether the failure of the autophagic clearance mechanism for the ASC-speck in microglia is part of a global failure in autophagy that results in the accumulation of AD/PD related proteins in neurons in these neurodegenerative diseases needs to be further explored. Moreover, if there is a common node for failure of the autophagy-lysosomal clearance pathway in both neurons and microglia, identifying this will be important for development of targeted therapeutics.

### Microglial Polarization Can Determine Proteinopathy Disease Progression or Clearance

Microglia generally exist in two major states: a “resting” state during which the microglia primarily perform an active surveillance role, and an “activated” state where microglia can perform a number of immune related tasks (Yao and Zu, 2020). The function performed by the “activated” microglia is dependent upon a process called polarization where the microglial cell is induced into a number of phenotypes with specific roles. The classical division are the M1 and M2 phenotypes, with a number of M2 subtypes playing various maintenance roles. In general, the M1 phenotype carries out pro-inflammatory behaviors while the M2 phenotype has an anti-inflammatory/pro-phagocytic role (Yao and Zu, 2020). In neurodegenerative disease, the role of M2 microglia in early stages of the disease has a neuroprotective role. However, as the disease progresses, prolonged and excessive activation of M1 microglia generates the neuroinflammatory state that exacerbates pathological damage and neuronal cell death (Floden and Combs, 2011). Our current understanding of microglial polarization with regards to AD has been reviewed by Yao and Zu (2020).

With the emergence of powerful single cell and single nucleus RNA sequencing allowing for profiling of microglia, the concept of M1, M2 and acquired deactivation phenotype of microglia is fading away. Based on single cell RNA sequencing, previous studies have identified a subset of microglia called “disease-associated microglia” (DAM), which show downregulation of *CX3CR1, P2RY12, CD33*, and *TMEM119*; and upregulation of *TYROBP, APOE, B2M*, and *TREM2* and appear to contribute to the disease process (Butovsky and Weiner, 2018; Deczkowska et al., 2018). There is an interesting subset of DAMs driven by Triggering Receptor Expressed on Myeloid Cells 2 (TREM2)-signaling that specifically sense damage within the CNS called “neurodegeneration-associated molecular patterns” (NAMPs), tailored to contain/remove CNS damage and provide homeostasis (Deczkowska et al., 2018). NAMP-responsive DAMs appear to play a protective role in neuroinflammatory diseases under some conditions and an exacerbating role in other conditions (Butovsky and Weiner, 2018; Deczkowska et al., 2018). It is unclear what may determine this conflicting function, but timing and disease progression may contribute to this duality (Mathys et al., 2017). It is unclear if the specific signaling that trigger NAMP-responsive DAMs are also responsible for preventing microglial ASC speck/inflammasomes from undergoing clearance via ubiquitin-proteosome system or autophagy-lysosomal pathways, or how the ASC speck/inflammasomes may play a role in dysregulation of the DAM phenotype contributing to loss of protective function.

### ASC Speck Release May Be an Attempt to Recruit Help to Clear Large Aggregates

The release of ASC specks into the extracellular environment, including during pyroptosis, in response to misfolded protein aggregates may be an attempt to recruit more phagocytic cells to clear these protein aggregates. Broderick and Hoffman (2014) speculated that ASC speck release into the extracellular environment in response to urate crystals that are too large to be phagocytosed by macrophages may serve as a signal amplification mechanism to recruit the assistance of neighboring cells in the immune response. Indeed, microglia that undergo inflammasome activation in response to Aβ aggregates release chemotactic factors for the recruitment of neighboring cells (Halle et al., 2008). Brain sections of AD patients commonly show activated microglia clustered around Aβ plaques (Nichols et al., 2019) as well as in apposition to pathological tau structures (Bolós et al., 2015). Similarly, in PD, a reactive microgliosis can typically be seen in brain regions with substantial α-syn pathology (Sanchez-Guajardo et al., 2015). Yet, it remains a mystery why innate immune cells may hinge upon ASC speck-mediated amplification despite the prevailing use of the cytokine/chemokine system for trafficking and recruitment of leukocytes/myeloid cells. Perhaps, such a scenario may occur primarily to only amplify IL-1β/IL-18 signaling in the context of a specific disease stage.

## The Role of the ASC Speck in the Accumulation and Spreading of Alzheimer’s Disease/Parkinson’s Disease Related Misfolded Protein Aggregates

### The ASC Speck Induces Pro-aggregatory Post-translational Modifications of Tau and α-Synuclein

NLRP3 inflammasome activity has been shown to be directly attributable to the pathogenesis of some of the misfolded protein aggregates in neurodegenerative diseases. Caspase-1 activity has been shown to cleave α-syn (at Asp121) to a highly aggregation prone form (Wang et al., 2016). Studies have also shown that caspase-cleaved tau (at Asp421) can also become prionoid and seed tau aggregation (Cotman et al., 2005) and can activate microglia (Cotman et al., 2005; Zilka et al., 2012) and thus drive the disease. We have previously shown that loss of tau can prevent neurodegeneration in a lipopolysaccharide (LPS) model of systemic inflammation in *Cx3cr1^–/–^* mice (Maphis et al., 2015a). Recently, we showed that doxycycline- or vaccine-mediated suppression of pathological tau blocks NLRP3, ASC, and IL-1β expression in microglia (Jiang et al., 2021).

NLRP3 inflammasome activity is also indirectly attributable to the pathogenesis of AD and PD related protein aggregates through inflammatory signaling-mediated upregulation of pro-aggregatory PTMs of tau and α-syn. Inflammatory signaling, such as through caspase cleaved IL-1β, upregulates neuronal kinase activity resulting in tau hyperphosphorylation and has been reviewed in Barron et al. (2017). Upregulation and activation of p38 mitogen-activated protein kinase (MAPK) (Li et al., 2003; Kitazawa et al., 2005), cyclin-dependent kinase 5 (CDK5) (Kitazawa et al., 2005; Roe et al., 2011), Glycogen Synthase Kinase 3 Beta (GSK-3β) (Kitazawa et al., 2005; Roe et al., 2011; Sy et al., 2011), *JNK* (Kitazawa et al., 2005; Roe et al., 2011), and extracellular signal-regulated kinase (ERK2) (Roe et al., 2011) have all been implicated in the pro-aggregatory hyperphosphorylation of various tau residues in response to inflammatory signaling.

The link between inflammatory signaling and pro-aggregatory PTMs of α-syn is not as well established, though one study demonstrated that LPS induced inflammation triggers α-syn phosphorylation at S129 and accumulation of α-syn aggregates that spread from the olfactory bulb to the substantia nigra and striatum resulting in PD-like pathology (Niu et al., 2020). The level of systemically circulating phosphorylated α-syn also has a positive correlation with the levels of NLRP3 and IL-1β in human PD patients (Chatterjee et al., 2020). There are numerous PTMs that promote the aggregation of α-syn (reviewed in Zhang et al., 2019) such as Casein kinase 2 (CK2)-mediated (Fujiwara et al., 2002; Smith et al., 2005) and G Protein-Coupled Receptor Kinase 2 (GRK2)-mediated (Feany and Bender, 2000) phosphorylation at S129, Seven *in absentia* homolog (SIAH)-mediated ubiquitination (Lee et al., 2008; Rott et al., 2008), Protein Inhibitor of Activated STAT 2 (PIAS2)-mediated SUMOylation (Rott et al., 2017), and nitration (Hodara et al., 2004). Whether IL-1β signaling directly contributes to these PTMs needs further investigation. Together these studies suggest a crosstalk between the NLRP3 inflammasome/ASC speck and neuron-derived protein aggregates in AD and PD.

### The ASC Speck as a Scaffold for Protein Aggregation

Based on such crosstalk, it is conceivable that the inflammasome proteins and the AD/PD protein aggregates may cross-seed. Sahillioğlu and Özören (2015) reported that ASC specks have an intrinsic property to co-aggregate cytosolic proteins on their surface through non-specific hydrophobic interactions. They speculated that this may indicate a role for ASC specks as supramolecular platforms for antigen presentation in innate immune cells during intracellular infection. They used HEK293T cells expressing m-Cherry-ASC to detect ASC speck formation and co-localization of the ASC speck with a panel of enhanced green fluorescent protein (EGFP)-fused short peptides, including the complement protein C3 as well as the model antigen ovalbumin. They found a diversity of cytosolic proteins that could co-aggregate on the ASC speck that primarily indicated non-specific hydrophobic interactions. They further speculated that antigen presentation on the ASC speck may allow for cross-presentation of antigens on MHC-II. Cytoplasmic proteins are mostly displayed by MHC-I, however, cytoplasmic proteins that enter the autophagic pathway are presented on MHC-II. Using phorbol-12-myristate-13-acetate (PMA)-differentiated THP-1 macrophages, they observed that purified extracellular ASC specks are engulfed by the phagocytic cell and contained within an acidified organelle, likely the phagolysosome, where it is then degraded over time and trafficked away in small tubular vesicles consistent with the morphology of tubular vesicles responsible for MHC-II dependent presentation of extracellular antigens (Sahillioğlu and Özören, 2015). ASC speck-dependent cross presentation of antigens on MHC-II has not been confirmed. Ciccocioppo et al. (2020) commented that misfolded protein aggregates look like PAMPs and thus activate a range of PRRs to activate the innate immune response in an attempt to clear the inciting stimulus, and when this response fails to clear the aggregates, the immune response remains locked in a toxic pro-inflammatory cascade.

#### Aggregation of Aβ by the ASC Speck

Heneka et al. (2013) demonstrated a clear link between NLRP3 inflammasome activity and Aβ-related pathology and its clearance in AD. They demonstrated that NLRP3 deficient APP/PS1 mice show no caspase-1 cleavage of pro-IL-1β and had IL-1β levels similar to non-transgenic control mice (Heneka et al., 2013). NLRP3^–/–^ and Caspase-1^–/–^ APP/PS1 mice show improved delay-dependent and spatial memory relative to APP/PS1 with intact NLRP3 inflammasome pathways. Hippocampal synaptic plasticity as measured by long term potentiation (LTP) was also protected in NLRP3^–/–^ and Caspase-1^–/–^ APP/PS1 mice relative to the LTP suppression observed in APP/PS1 mice (Heneka et al., 2013). NLRP3 deficient mice were also protected from neurobehavioral disturbances as demonstrated by open field testing. NLRP3^–/–^ and Caspase-1^–/–^ APP/PS1 mice had reduced Aβ plaque load in both hippocampus and cortex even though levels of APP expression and processing by β-secretase-1 were unaffected suggesting that the decrease in aggregated Aβ was due to enhanced clearance mechanisms. Furthermore, they clearly demonstrated that NLRP3^–/–^ and Caspase-1^–/–^ APP/PS1 had enhanced phagocytosis of amyloid-β plaques with evidence of degradation by lysosomes. There was also evidence of enhanced insulin-degrading enzyme release by microglia in inflammasome deficient APP/PS1 mice. They also characterized microglial phenotype and found that NLRP3 and caspase-1 deficiency resulted in a skewed microglial phenotype toward the pro-phagocytic M2 phenotype. The upregulation of nitric oxide synthase-2 in M1 microglia in APP/PS1 mice results in nitration of tyrosine on Aβ accelerating its aggregation and seeding of new plaques. NLRP3^–/–^ and Caspase-1^–/–^ APP/PS1 had significantly less nitrated Aβ as well as smaller plaque sizes (Heneka et al., 2013). This study demonstrated that there is a clear link between NLRP3 inflammasome/ASC speck activation and AD progression, especially regarding the accumulation of Aβ plaques. Furthermore, NLRP3 inflammasome/ASC speck activation contributes to a pro-inflammatory microglial phenotype that reduces protein clearance mechanisms.

Venegas et al. (2017) demonstrated that extracellular ASC specks directly cross-seed Aβ aggregation *in vitro* and *in vivo*. Exposure of cultured mouse primary microglia to Aβ_1–42_ caused the formation and release of ASC specks which were shown to associate with TAMRA-labeled Aβ_1–42_ very rapidly in the extracellular environment (Venegas et al., 2017). Incubation of the supernatant derived from non-transgenic mouse primary microglia stimulated with LPS and ATP to produce inflammasomes with Aβ_1–42_ resulted in detectable Aβ_1–42_ aggregation while ASC-deficient microglia did not (Venegas et al., 2017). Purified ASC specks incubated with Aβ_1–42_ accelerated aggregation speed in a concentration-dependent manner. The decreased lag phase of Aβ_1–42_ aggregation in the presence of the ASC speck indicates a cross-seeding ability of the ASC speck through its interactions with the Aβ_1–42_ peptide. Interestingly, this cross-seeding ability was specific to the Aβ_1–42_ peptide and did not occur with the reverse sequence of Aβ_1–42_ or with the model protein bovine serum albumin (BSA). Co-sedimentation assays in which the insoluble pellet fraction is separated from the cell supernatant, and both are analyzed to determine the presence of Aβ and ASC, showed that ASC-specks alone remain in the supernatant but when incubated with Aβ_1–42_ or Aβ_1–40_ will co-sediment into the pellet fraction together (Venegas et al., 2017). The *in vitro* co-sedimentation findings were corroborated by immunoprecipitation studies *ex vivo* in brain samples from APP/PS1 mice (Venegas et al., 2017). Immunohistochemical studies revealed that ASC specks were found in the core of extracellular Aβ plaques in APP/PS1 mouse models as early as 4-months as well as in human AD patients including the early mild cognitive impairment stages of AD (Venegas et al., 2017). APP/PS1-ASC^–/–^ mice showed reduced Aβ plaque deposition and had reduced spatial memory deficits as assessed by Morris water maze. Purified ASC specks that were injected into the hippocampus of 3-month-old APP/PS1 mice showed increased size and number of Aβ plaque deposition relative to the contralateral non-injected side without changes in phagocytosis, amyloid-precursor protein (APP) production, or a-/b-C-terminal fragments of APP (Venegas et al., 2017). APP/PS1-ASC^–/–^ mice injected with brain homogenates from aged APP/PS1 mice in the hippocampus showed significantly reduced size, amount, and spreading of Aβ plaques relative to APP/PS1 mice that were not deficient in ASC (Venegas et al., 2017). This could not be explained by changes in Aβ degradation. Similarly, APP/PS1 injected with brain homogenates from ASC^–/–^ APP/PS1 mice showed reduced Aβ plaque deposition. These results demonstrate that the ASC speck can serve as a scaffold for cross-seeding of Aβ aggregation even in very early stages of AD and thus plays a role in the progression and spreading of Aβ pathology in the brain.

#### Aggregation of α-Synuclein by the ASC Speck

Similar to AD, human PD patients have elevated NLRP3, ASC, and caspase-1 localized exclusively within Iba1^+^ microglia in the substantia nigra or detectable as extracellular ASC and systemically circulating NLRP3, caspase-1, and IL-1β (Gordon et al., 2018; Chatterjee et al., 2020). Wang et al. (2016) demonstrated that caspase-1 can be found at the core of Lewy bodies extracted from human PD patients’ brains. In this study, they stained Lewy bodies for caspase-1 and α-syn and showed a caspase-1 positive core (∼10 microns in diameter) surrounded by α-syn (Wang et al., 2016). While they did not stain for ASC to determine if the caspase-1 positive core was an ASC speck, based on the size of the core and what is known about the ASC speck’s ability to cross-seed protein aggregation, we speculate that ASC specks can likely be found in the core of α-syn Lewy bodies similarly to Aβ plaques.

Additionally, Gordon et al. (2018) used three animal models of PD to study inflammasome related dopaminergic neurodegeneration that is driven by mitochondrial dysfunction, oxidative stress, or α-syn pathology. These animal models used 6-hydroxydopamine (6-OHDA) administration, MitoPark transgenic mice, and pre-formed α-syn fibril (PFF) injected mice to induce PD (Gordon et al., 2018). In each animal model, NLRP3, ASC, and caspase-1 were elevated at early stages, even prior to α-syn fibril formation, confirming that NLRP3 inflammasome activity can be stimulated by α-synuclein oligomers or protofibrils and can contribute to α-syn fibril deposition. Primary mouse microglia incubated with α-syn PFFs exhibited robust NLRP3 inflammasome activation that was delayed relative to ATP stimulation (24 h vs. 1 h) likely because inflammasome activation by fibrils cannot occur until the fibrils are phagocytosed and escape lysosomal degradation (Gordon et al., 2018). They also observed significant levels of extracellular ASC from primary microglia exposed to α-syn PFFs despite not detecting any pyroptosis, suggesting that ASC specks may be released in a non-cell death-dependent manner, possibly via exosomal excretion. α-syn PFFs also activated primary microglia without any priming, suggesting that α-syn fibrils can act at signal 1 and signal 2, though the IL-1β release in unprimed cells were only ∼35% of the levels released in cells that were primed with LPS (Gordon et al., 2018). Injection of fibrillar α-syn in the striatum of WT mice results in deposition of pathological hyperphosphorylated α-syn aggregates in neurons and microglia of the nigrostriatal system as well as in the cortex (Gordon et al., 2018).

Another group used a chronic neurotoxicant, 1-Methyl-4-phenyl-1,2,3,6-tetrahydropyridine (MPTP), model of PD and found that NLRP3 deficiency protected against nigral dopaminergic neurodegeneration, prevented microgliosis and astrogliosis in the substantia nigra and prevented α-syn aggregate deposition in the substantia nigra (Ou et al., 2021). NLRP3 deficiency also protected against MPTP induced impairments in nigral autophagy pathways in the midbrains of mice as measured by the expression of the autophagy related proteins, LC-II and p62 (Ou et al., 2021). It is unclear if this failure in autophagy is cell-specific (neurons vs. microglia) or global. These studies suggest a similar crosstalk between a-syn aggregates and inflammasomes in the cross-seeding process in PD to what was demonstrated with Aβ in AD.

#### Aggregation of Tau by the ASC Speck

Previous studies have also demonstrated a link between inflammasome/microglial inflammatory activity and tau neurofibrillary tangle (NFT) seeding/accumulation in rodent models of tauopathy. Ising et al. (2019) showed that NLRP3 activation is elevated in FTD patients as evidenced by elevated ASC, caspase-1, and mature IL-1β levels. Additionally, Tau22 mouse models of FTD that were deficient in ASC (ASC^–/–^) or NLRP3 (NLRP3^–/–^) had reduced levels of phosphorylated tau in the hippocampus, CA1 cell body region, and granular cell layer of the dentate gyrus as assessed by AT8 staining; they also had decreased aggregated tau levels as assessed by Thioflavin T fluorescence assays; and they exhibited rescue in spatial memory relative to Tau22 mice that had intact NLRP3 inflammasome pathways (Ising et al., 2019). In Stancu et al. (2019), TauP301S (PS19) transgenic mice deficient in ASC (ASC^–/–^) were injected with pre-aggregated tau seeds in the frontal cortex at 3 months of age. The results showed significantly decreased seeding ability of tau relative to PS19 mice that were not deficient in ASC indicating a significant role for the inflammasome in tau propagation in an ASC-dependent manner (Stancu et al., 2019). Another group previously demonstrated that microgliosis and microglial activation precede tau neurofibrillary tangle burden in PS19 mice and treating PS19 with an anti-inflammatory molecule, FK506, significantly reduced tau pathology and extended the life-span of PS19 mice (Yoshiyama et al., 2007). We have previously demonstrated that adoptive transfer of purified microglia from hTau-*Cx3cr1*^–^*^/^*^–^ mice that exhibit a highly inflammatory phenotype, into the brains of non-transgenic recipient mice was sufficient to induce tau pathology in a manner dependent upon IL-1β-IL-1R axis (Maphis et al., 2015b). In our recent study, we have demonstrated that myeloid-cell restricted deletion of MyD88 blocked both signal 1 (priming) and signal 2 (inflammasome activation) and myeloid-cell specific deletion of ASC prevented (signal 2) for NLRP3 inflammasome activation and, in both cases, it reduced maturation of IL-1β, reduced tau pathology, and improved memory in the hTau mouse model of tauopathy (Jiang et al., 2021).

A previous study by Asai et al. (2015) showed that depletion of microglia and inhibition of exosome synthesis mitigated the propagation of tau and tau spreading across brain regions. Much of their definitive work was performed in a model using an adeno-associated virus vector expressing human P301L mutant tau in a neuron-specific manner, by injecting it into the entorhinal cortex of C57BL/6j mice. Intracortical inject of P301L FTDP-17 tau exhibited rapid propagation of tau from the entorhinal cortex to the dentate gyrus in 4 weeks. They also corroborated their findings using TauP301S mice. This study focused on the role of tau-containing exosome release from microglia as a method for tau spreading, which is another important way that microglia contribute to AD/PD disease progression, though not the focus of this review. The depletion of microglia using PLX3397, a colony stimulating factor 1 receptor inhibitor, in P301S mice demonstrated reduced AT8 (pS202/pT205 positive) tau burden at 3.5 months of age which supported their claims that microglia play an important role in the spreading of protein aggregates and disease progression (Asai et al., 2015). We speculate that depletion of microglia using PLX3397 may have also reduced ASC speck-mediated aggregation of tau in addition to exosomal spreading, though this was not investigated. Though we will not address them in this review, recent studies have also indicated that the NLRP3 inflammasome may play an important role in promoting exosome release from microglia (Si et al., 2021).

**FIGURE 3:**
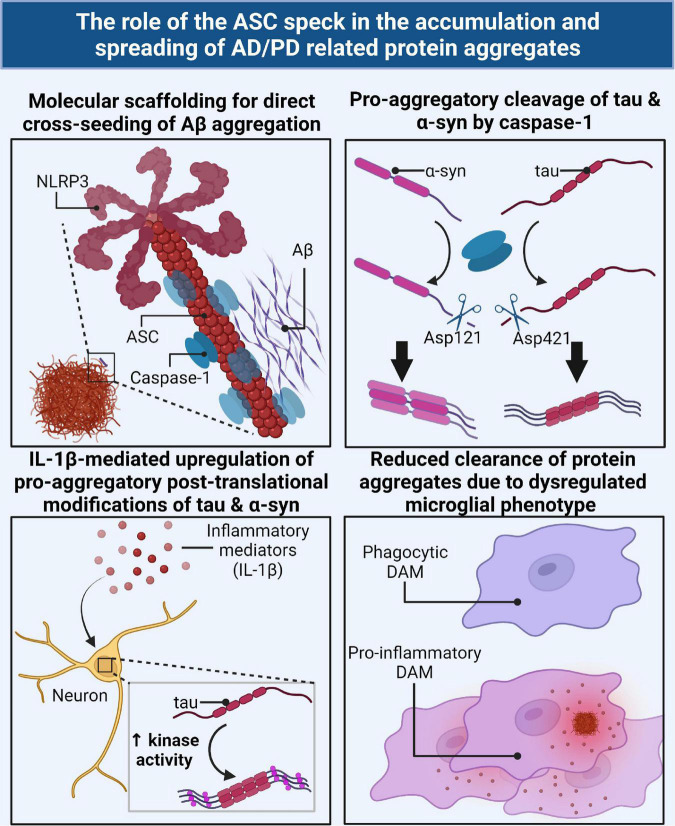
The ASC speck directly and indirectly contributes to the accumulation and spreading of AD/PD related misfolded protein aggregates via several mechanisms. The ASC speck can directly cross-seed the aggregation of Aβ by serving as a molecular scaffold for aggregation **(top left)**. Caspase-1, the effector component of the inflammasome/ASC speck, can directly cleave tau and α-syn monomers into a pro-aggregatory form for seeding nucleation **(top right)**. IL-1β, the downstream product of the ASC speck, can act as a cellular signal to upregulate key enzymes for the pro-aggregatory PTMs of tau and α-syn **(bottom left)**. The ASC speck contributes to the dysregulation of protective disease-associated microglia (DAMs) and subsequent skewed pro-inflammatory phenotype resulting in a decrease in phagocytic clearance of AD/PD related misfolded protein aggregates **(bottom right)**.

Taken together, these results suggest that microglia can promote seeding/spreading of numerous AD/PD related misfolded protein aggregates as well as pathological modifications of tau and a-syn with a multitude of different mechanisms (Figure 3). Importantly, inflammasome activity and/or assembly of the ASC speck underscores many of these mechanisms.

### Inhibitors of NLRP3 or Antibody-Neutralization of Inflammasomes Reduce Disease Burden

Given its apparent role in AD and PD pathogenesis and disease progression, the NLRP3 inflammasome pathway has become a target for intervention. Several studies have used small molecule inhibitors of NLRP3 to prevent inflammasome assembly and seen significant reductions in inflammation as well as rescue of cognitive function in animal models of AD and PD. Use of targeted antibody therapies against inflammatory signaling molecules and complexes, such as the ASC speck, is also a growing area of therapeutic development. In this section, we will highlight recent studies that have targeted the NLRP3 inflammasome pathway using these therapeutic strategies.

Dempsey et al. (2017) showed reduced Aβ plaque burden, reduced inflammation, and rescue of cognitive function in the APP/PS1 animal model of AD using the NLRP3 inhibitor, MCC950. Cultured primary microglia from C57B/L6 mice were incubated with LPS and ATP or LPS and Aβ for NLRP3 activation. Pre-incubation with MCC950 attenuated the production of IL-1β in both groups as well as caspase-1 immunoreactivity (Dempsey et al., 2017). Only the LPS + ATP group exhibited lactate dehydrogenase release indicating loss of cell viability, but this was attenuated by MCC950. As expected, MCC950 had no effect on TNFα or IL-6 levels, which are expressed independent of inflammasome activation. MCC950 administration in primary microglia incubated with LPS and Aβ showed enhanced phagocytosis of Aβ relative to untreated microglia *in vitro*. These findings were confirmed *in vivo* using the APP/PS1 mouse model (Dempsey et al., 2017). Treatment of 12-month-old APP/PS1 mice with MCC950 every second day for 3 months showed a decrease in plaque number and soluble Aβ_1–40_,_1–42_ suggesting increased phagocytic activity, though various indicators of microglial phenotype did not reach significance to indicate promotion of an M2 phenotype. The anti-inflammatory and pro-phagocytic effects of MCC950 administration in APP/PS1 mice correlated with improvement in cognitive function as assessed by spontaneous alternation in the T-maze and the novel object recognition test (Dempsey et al., 2017).

Another study using CRND8 APP transgenic mice aged 9 months injected with a novel NLRP3 small molecule inhibitor, JC-124, reduced levels of caspase-1 relative to untreated transgenic mice, reduced the size and number of Aβ oligomers and plaques, and reduced β-secretase cleavage of APP (Yin et al., 2018). Microgliosis was reduced by JC-124 in TgCRND8 mice but a concomitant astrocytosis was observed. Reduction in oxidative stress was also observed following treatment with JC-124 as assessed by the oxidative stress markers heme oxygenase-1 (HO-1) and 4-hydroxynonenal (HNE). There were also improvements in synaptophysin levels suggesting a synaptoprotective effect of JC-124 in TgCRND8 mouse models (Yin et al., 2018).

To confirm the role of the ASC speck in the cross-seeding of Aβ protein aggregates, Venegas et al. (2017) used antibody neutralization studies against the ASC speck. Co-incubation of an anti-ASC antibody with purified ASC-specks and Aβ prevented ASC speck-induced aggregation of Aβ in an antibody concentration dependent manner (Venegas et al., 2017). This was confirmed *in vivo* by co-injecting APP/PS1 hippocampi with brain homogenates from aged APP/PS1 mice with either anti-ASC antibodies or an isotype-specific IgG. The results showed that anti-ASC antibodies reduced the rostral-caudal spreading of Aβ plaque deposition as well as reduced Aβ monomers and oligomers without affecting the APP production or degradation pathways (Venegas et al., 2017). Thus, antibodies against the ASC speck may be a useful strategy for inhibiting its cross-seeding potential and for mitigating the inflammatory response in AD, which may also likely be effective in PD.

In the context of tauopathy, MCC950 administration in LPS-primed primary microglia exposed to pre-aggregated tau seeds prevented inflammasome assembly and IL-1β maturation (Stancu et al., 2019). MCC950 administration in PS19 transgenic mice injected with pre-aggregated tau seeds reduced exacerbation of tau pathology as assessed by AT8 staining in a dose dependent manner as well as microgliosis as assessed by Iba1 staining (Stancu et al., 2019). It was noted that reduction in microgliosis could either be due to reduction in inflammasome assembly (or ASC speck formation) and thus reduced inflammatory cytokine signaling, or reduction in tau pathology, or a combination of the two (Stancu et al., 2019).

In the context of PD, MCC950 blocked NLRP3 inflammasome activity including ASC fibril formation in response to α-syn PFF aggregates in primary mouse microglia (Gordon et al., 2018). MCC950 also blocked inflammasome activation in the 6-OHDA, MitoPark, and PFF models of PD (Gordon et al., 2018). Daily oral dosing of MCC950 in the acute toxicant 6-OHDA model of PD also protected against nigrostriatal dopaminergic degeneration and behavioral deficits *in vivo* (Gordon et al., 2018). The same findings were observed in the chronic progressive PFF mouse model of PD in which chronic dosing of MCC950 prevented progressive motor and behavioral deficits as well as dopaminergic degeneration in response to pathological α-syn aggregates *in vivo* (Gordon et al., 2018). Chronic dosing of MCC950 also prevented the pathological spreading of α-syn aggregates from the PFF injection site in the striatum throughout the nigrostriatal system as well as into cortical brain regions (Gordon et al., 2018). This suggests that NLRP3 inflammasome activity is central to the propagation and spreading of α-syn throughout the brain in PD and can thus be targeted with therapeutic strategies.

Several therapeutics that act upstream of inflammasome activation to inhibit NLRP3 have all shown therapeutic effects in animal models of PD. Han et al. (2019) used kaempferol, a small molecule that inhibits NLRP3 inflammasome activity by promoting the ubiquitin-mediated autophagic degradation of NLRP3, and demonstrated reduced behavioral deficits, reduced nigral dopaminergic neurodegeneration, and enhanced tyrosine hydroxylase activity in an LPS-injected mouse model of PD as well as reduced dopaminergic neurodegeneration and microglial activation in an *A53T^tg/tg^* α-syn over-expressing mouse model of PD. Yao et al. (2019) used fingolimod (FTY720), an upstream inhibitor of NLRP3 activation by reducing reactive oxygen species and p65-mediated activation of NLRP3, in the MPTP mouse model of PD and showed reduced behavioral deficits, reduced dopaminergic neurodegeneration, and increased dopamine release in addition to reduced levels of IL-1β, IL-6, and TNFα. Xu et al. (2019) had similar findings using DDO-7263, a novel Nrf2-ARE activator that inhibits NLRP3 inflammasome activation, in the MPTP mouse model of PD.

Even inhibiting the downstream components of the inflammasome pathway have shown some therapeutic effect in animal models of PD. Injections of the caspase-1 inhibitor, Ac-YVAD-CMK, into the brains of two rat models of Parkinson’s disease (LPS-induced and 6-OHDA induced) reduced neurodegeneration of dopaminergic neurons in the substantia nigra and reduced the expression of inflammasome components (Mao et al., 2017).

Taken together, these studies indicate that inhibition of the NLRP3 inflammasome/ASC speck at various stages of its pathway (inhibiting inflammasome assembly, its downstream inflammatory signaling components, or the ASC speck’s interaction with other proteins) has potential to reduce or prevent disease progression in AD and PD.

## Discussion

**FIGURE 4:**
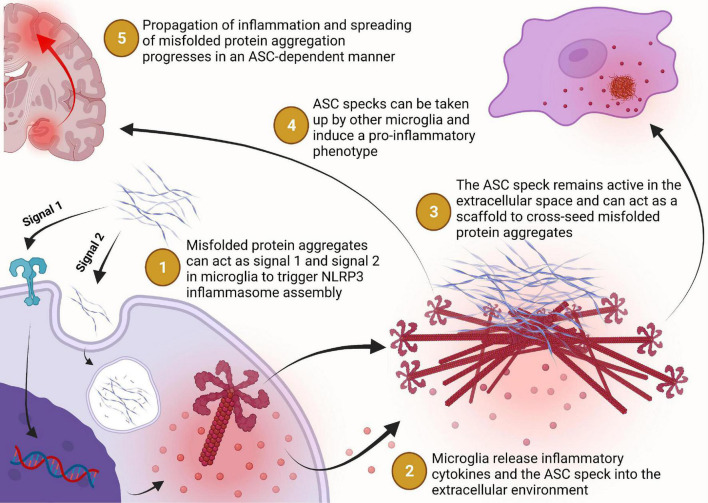
Misfolded protein aggregates bind to toll-like or scavenger receptors which triggers the expression of NLRP3 and caspase-1 to prime the cell for inflammasome assembly in a MyD88-NFκB signaling dependent manner. Extracellular misfolded protein aggregates may be internalized by the phagolysosomal pathway but can escape lysosomal degradation and trigger assembly of the NLRP3 inflammasome (step 1). The NLRP3 inflammasome catalyzes the maturation of interleukin-1β and interleukin-18 which can be transported extracellularly or released into the extracellular environment during pyroptosis for inflammatory signaling (step 2). Assembly of the NLRP3 inflammasome may progress to the formation of the ASC speck, a supramolecular complex with enhanced inflammatory signaling capacity, which may then be released into the extracellular environment following pyroptosis or some other non-pyroptic mechanism (step 2). Once in the extracellular space, the ASC speck remains functionally active for inflammatory cytokine maturation, pro-aggregatory modifications of other proteins, and cross-seeding of neuronally derived misfolded protein aggregation (step 3). The ASC speck can also propagate inflammation from one microglial cell to another by inducing a pro- inflammatory phenotype in cells that phagocytose the ASC speck but fail to degrade it (step 4). Together, these activities of the ASC speck implicate its role in the propagation of inflammation and spreading of protein aggregation in a vicious cycle that contributes to the chronic progressive nature of Alzheimer’s disease and Parkinson’s disease (step 5).

We have known for some time now that inflammation is a core characteristic of neurodegenerative diseases. It was previously believed to be a byproduct of the toxicity of the misfolded protein aggregates but over the last decade there has been significant progress in understanding how inflammation plays a major role in driving the disease. Within the last 5 years, several groups have begun to elucidate the active role that the NLRP3 inflammasome and ASC speck play in exacerbating misfolded protein aggregation and damaging neuroinflammation that drives disease progression and spreading throughout the brain (Figure 4). Thus, paving the way to a new paradigm in inflammasome biology that has broadened our understanding of the pathogenesis of neurodegenerative diseases like AD and PD. There are still many unanswered questions surrounding this new paradigm and many new opportunities for therapeutic development to be explored.

It seems that the ASC speck can cross-seed Aβ aggregation directly by acting as a molecular scaffold for aggregation and plays an important role in triggering the aggregation of tau and α-syn through a variety of mechanisms including upregulation of pro-aggregatory PTMs. This prion-like activity is particularly interesting because the ASC speck does not have the same β-pleated sheet fibrillar structure that is required for aggregation of the AD/PD related protein aggregates. Conversely, these misfolded protein aggregates can prime and activate the aggregation of NLRP3 inflammasome components into the prion-like ASC speck. It is unclear mechanistically how these protein aggregates trigger the activation of the NLRP3 inflammasome complex, but it is fascinating that there seems to be bi-directional cross seeding that triggers prionoid protein aggregation.

PTMs of the proteins involved in the inflammasome complex is important for regulating inflammasome assembly in response to various cellular stress signals as well as the decision to assemble the supramolecular ASC speck complex. The advances in single cell and single nucleus RNA sequencing that have allowed for the profiling of NAMP-responsive DAMs in AD and PD will likely permit further understanding of how misfolded protein aggregates contribute to the cellular decision to commit to the formation of the ASC speck.

Failed degradation of the ASC speck by microglial autophagy pathways results in the persistence of the active ASC speck contributing to an unregulated inflammatory response. This seems to coincide with a failure in the degradation of AD/PD related misfolded protein aggregates in neurons. Further research is necessary to understand why these degradation pathways fail in neurodegenerative diseases and to understand if there is a common link in the failure of both microglial and neuronal protein aggregate degradation pathways. Understanding how this pathological process occurs will be useful for identifying ways to generate therapeutics for disease prevention and intervention.

Therapeutics targeting the NLRP3 inflammasome and ASC speck have shown promising results in animal models of AD and PD. A multi-pronged approach targeting inflammasome/ASC speck assembly and activity, misfolded protein aggregates, and protein clearance mechanisms will likely be the future direction for therapeutic design in the treatment of neurodegenerative proteinopathies like AD and PD. With this new understanding of mechanisms contributing to disease progression, the future for generating effective treatments in the clinical management of AD and PD seems hopeful.

## Author Contributions

JH performed the literature search and drafted the manuscript. KB edited the manuscript. Both authors contributed to the article and approved the submitted version.

## Conflict of Interest

The authors declare that the research was conducted in the absence of any commercial or financial relationships that could be construed as a potential conflict of interest.

## Publisher’s Note

All claims expressed in this article are solely those of the authors and do not necessarily represent those of their affiliated organizations, or those of the publisher, the editors and the reviewers. Any product that may be evaluated in this article, or claim that may be made by its manufacturer, is not guaranteed or endorsed by the publisher.
